# Challenges in Conceptualising Modern Masculinity in Adolescent Mental Health Research

**DOI:** 10.1192/bjo.2026.11223

**Published:** 2026-06-30

**Authors:** Isobel Cammish, Mina Fazel

**Affiliations:** University of Oxford, Oxford, United Kingdom

## Abstract

**Aims:**

Adolescent boys experience high rates of suicide, substance-related harm and school exclusion compared to other genders. However, large-scale studies report lower prevalence of common mental health disorders in boys, raising concern that boys’ mental distress is under-recognised in research and clinical settings. We aimed to examine how constructions of masculinity influence the expression, recognition and measurement of mental distress in adolescent boys aged 10–16. We explored whether current research tools adequately capture male-typical presentations of mental distress, with implications for psychiatric research, clinical assessment and service provision.

**Methods:**

A critical narrative synthesis approach was used, using research from adolescent psychiatry, men’s health and public health literature. Evidence from large population-based studies of young people was considered alongside qualitative and epidemiological studies looking at gendered patterns of symptoms, help-seeking behaviour and clinical recognition. Masculinity was conceptualised as a socially constructed and context-dependent set of norms that interact with biological and structural factors. Disordered eating and body image concerns were used as a focused case study to illustrate how male-typical expressions of distress may not be captured by common questionnaires used clinically and in research.

**Results:**

The literature suggests that adolescent boys are more likely to express mental distress through externalising behaviours, irritability, risk-taking, and body-focused practices, which are less likely to be identified by commonly used mental health questionnaires. Survey items frequently reflect female-typical symptom profiles and ideals when assessing body dysmorphia and disordered eating, potentially contributing to an under-detection of eating disorders in boys. Masculine norms around emotional restriction, physical strength and self-reliance may influence how boys experience, interpret and report symptoms, while professional biases and service structures can limit recognition and care when boys do seek help. These factors contribute to a cycle of poor identification, research support, and outcomes.

**Conclusion:**

Current gender-blind measurements of adolescent mental health risk systematically under estimate clinically significant mental distress in adolescent boys. Greater attention to how masculinity affects the expression, reporting and identification of psychiatric symptoms is needed to improve the accuracy of research and psychiatric assessment to ensure boys’ needs are adequately recognised. Incorporating male-typical presentations into screening tools and engaging boys in the design of research measures may improve how boys’ mental health is conceptualised and measured. Addressing these gaps has significant implications for child and adolescent mental health services, prevention strategies and psychiatric training – which has the potential to benefit not only boys, but everyone whose distress does not align with typical diagnostic criteria.

